# Chemically Modified Walnut Shell Biochar as a Sustainable Filler for Natural Rubber: Surface Properties, Filler–Filler Interactions and Mechanical Performance

**DOI:** 10.3390/ma19194224

**Published:** 2026-10-05

**Authors:** Justyna Miedzianowska-Masłowska, Marcin Masłowski, Wiktor Wyderkiewicz

**Affiliations:** Institute of Polymer and Dye Technology, Lodz University of Technology, Stefanowskiego 16, 90-537 Lodz, Poland; wiktor.wyderkiewicz@dokt.p.lodz.pl

**Keywords:** biochar, natural rubber, surface functionalization, sustainable filler, polymer composites

## Abstract

Walnut-shell biochar (BC) was investigated as a sustainable filler for natural rubber (NR), with particular emphasis on the effects of chemical purification and subsequent surface functionalization on the properties of the resulting vulcanizates. BC was treated with citric acid (CA) or sodium hydroxide (NaOH) and subsequently functionalized using 3-aminopropyltriethoxysilane (APTS) or cetyl alcohol (CAl). The applied treatments substantially modified the surface chemistry and mineral composition of BC, as confirmed by FTIR spectroscopy and elemental analysis. Incorporation of BC increased the tensile strength of NR from 14.1 to 22.1 MPa and increased the crosslink density from 2.35 × 10^−5^ to 2.84 × 10^−5^ mol cm^−3^. Chemical purification also markedly affected the vulcanization behavior; the NaOH-treated BC increased the torque increment to 4.55 dNm and reduced the optimum cure time to 1.50 min, compared with 2.88 dNm and 1.99 min for the unfilled NR, respectively. Surface functionalization further modified the network structure, with the highest crosslink density of 3.45 × 10^−5^ mol cm^−3^ obtained for the APTS-functionalized BC. Particularly pronounced effects were observed during thermo-oxidative aging, where the aging coefficient increased from 0.31 for NR containing untreated BC to 0.89 for the CA-treated and CAl-functionalized system. The Payne effect also depended strongly on the modification route, decreasing from 75.5% for untreated BC to 40.1% after NaOH treatment. Overall, the results demonstrate that chemical purification combined with appropriate surface functionalization provides an effective approach for tailoring walnut-shell BC–NR interactions, vulcanization behavior, mechanical performance and aging resistance.

## 1. Introduction

Growing demand for reducing the use of fossil-derived raw materials in polymeric materials has increased interest in fillers obtained from renewable and waste biomass [1,2]. In elastomer applications, particular attention has been given to carbonaceous bio-based fillers that could partially replace conventional carbon black [3]. Although carbon black remains one of the most effective reinforcing fillers, its production is predominantly based on non-renewable fossil resources. Biochar, a carbon-rich solid produced by the thermochemical conversion of biomass under limited oxygen conditions, represents a promising alternative that can simultaneously contribute to the valorization of agricultural and lignocellulosic waste [4,5].

The reinforcing performance of biochar depends not only on its carbon content but also on particle size, morphology, specific surface area, porosity, ash content and surface chemistry. Importantly, these characteristics vary considerably with the type of biomass and pyrolysis conditions. Consequently, biochar cannot yet be considered a direct drop-in replacement for carbon black, and tailoring its physicochemical properties is essential for achieving satisfactory reinforcement in rubber composites [6]. Several studies have nevertheless demonstrated its potential in elastomers. Biochar derived from *Paulownia elongata* was reported to partially replace carbon black in natural rubber while maintaining favorable mechanical properties [7], whereas lignin-derived biochar showed a reinforcing effect in styrene-butadiene rubber comparable to carbon black N330 at selected filler loading [8].

Among different biomass feedstocks, walnut shells are particularly attractive because they are an abundant agricultural by-product containing significant amounts of lignin, cellulose and hemicellulose. Their composition depends on the origin and preparation of the biomass; reported values include approximately 18–24% cellulose, 22–36% hemicellulose and 37–50% lignin [9,10]. Walnut shells also contain mineral components and extractive compounds, which can contribute to the heterogeneous surface chemistry of the resulting biochar. Their high lignin content is particularly relevant for thermochemical conversion because lignin favors the formation of a carbon-rich aromatic structure [11].

Walnut-shell-derived materials have already been investigated as biofillers in natural rubber [9]. More recently, walnut-shell biochar was demonstrated to be an effective reinforcing material for NR. Ball milling of the biochar increased the availability of surface functional groups and improved its reinforcing efficiency; at an optimized loading, the tensile strength of the biochar-filled vulcanizate approached that of an N330 carbon-black-filled reference [12]. These results confirm the potential of walnut shells as a feedstock for producing functional carbonaceous fillers for elastomer applications.

However, the heterogeneous nature of biochar may also limit its performance. Residual mineral components and oxygen-containing surface groups can affect filler–filler interactions, filler–rubber adhesion and the vulcanization process. In particular, the inorganic fraction may influence the local chemical environment of the curing system. Transition-metal ions such as Fe, Cu, Mn and Co can also promote oxidative processes in natural rubber, making the mineral composition of biomass-derived fillers an important factor when considering the long-term performance of rubber composites [13,14,15].

Chemical modification provides an opportunity to control these effects. Acidic and alkaline treatments can alter the mineral fraction, porosity and concentration of surface functional groups [16,17]. For example, alkaline treatment with NaOH has been reported to increase the specific surface area and pore volume of biochar, whereas acidic treatment can modify the concentration and character of surface acidic groups [18]. Such treatments may therefore influence not only the physicochemical properties of the filler but also its wettability and interactions with the rubber matrix.

Surface functionalization provides a further possibility for controlling the biochar–rubber interface. Organosilanes are widely used to modify filler surfaces and improve compatibility between fillers and polymer matrices [19,20,21]. In particular, 3-aminopropyltriethoxysilane (APTS) introduces amino-functional groups that may promote specific interactions with the surrounding matrix and alter the balance between filler–filler and filler–rubber interactions [22]. An alternative approach involves the use of long-chain fatty alcohols and their derivatives as filler surface modifiers, which can alter surface polarity, hydrophobicity and wettability through the presence of hydrophobic aliphatic chains [22]. These two approaches therefore represent different strategies for modifying the biochar–rubber interface.

Despite the growing interest in biochar-filled elastomers, relatively few studies have systematically investigated how chemical pretreatment followed by different surface-functionalization strategies affects the structure and performance of the same biochar in natural rubber. In particular, the relationships between surface chemistry, elemental composition, wettability, filler–filler interactions, crosslinking behavior and mechanical performance remain insufficiently established. Recent studies have demonstrated that the physicochemical properties of biochar are strongly dependent on the biomass feedstock, pyrolysis conditions and subsequent chemical activation or surface modification, which can substantially affect its chemical composition, thermal behavior and morphology [23]. These findings highlight the importance of considering the entire preparation and modification pathway when evaluating biochar as a functional filler for polymeric materials [24,25,26].

In this context, citric acid and NaOH were selected as chemical treatment agents to modify the chemical composition and surface characteristics of biochar, while 3-aminopropyltriethoxysilane (APTS) and cetyl alcohol (CAl) were selected as surface modifiers providing different approaches to altering surface functionality and polarity. However, the effects of combining chemical pretreatment with subsequent surface functionalization on the performance of walnut-shell biochar in natural rubber have not yet been systematically established.

The economic feasibility of walnut-shell biochar as a rubber filler is strongly dependent on the scale and conditions of the production process. Although the use of biomass waste as a feedstock provides an opportunity for waste valorization and the production of a renewable alternative to conventional mineral or fossil-derived fillers, the costs of pyrolysis, purification and surface functionalization can differ substantially between laboratory and industrial scales. An additional advantage of the proposed approach is the possibility of tailoring the biochar surface properties to the requirements of the rubber matrix through relatively simple chemical modification routes. A reliable economic assessment would therefore require process scale-up and a dedicated techno-economic analysis, which were beyond the scope of the present study.

Against this background, the novelty of the present study lies in the systematic evaluation of walnut-shell-derived biochar subjected to different chemical pretreatments (citric acid and NaOH) and subsequent surface functionalization with APTS or cetyl alcohol. The effects of these modifications were evaluated not only through mechanical properties but also by combining chemical and physicochemical characterization, wettability, rheological behavior, vulcanization characteristics, crosslink density and viscoelastic response, including the Payne effect. This comprehensive approach allows the relationship between biochar surface modification, filler–filler/filler–rubber interactions and the final performance of natural-rubber vulcanizates to be established. Such knowledge is important for the development of bio-based reinforcing fillers with tunable surface properties and for the valorization of agricultural waste in more sustainable elastomeric materials.

## 2. Materials and Methods

### 2.1. Materials

Natural rubber (NR), grade RSS1, based on cis-1,4-polyisoprene, was supplied by Torimex-Chemicals Ltd. Sp. z o.o. (Konstantynów Łódzki, Poland) and used as the elastomeric matrix. Zinc oxide (ZnO), sulfur (S), 2-mercaptobenzothiazole (MBT) and stearic acid (SA) were obtained from Sigma-Aldrich (St. Louis, MO, USA). Walnut shells were used as the lignocellulosic precursor for the preparation of biochar (BC). Citric acid (CA), sodium hydroxide (NaOH) and 3-aminopropyltriethoxysilane (APTS) were obtained from Sigma-Aldrich (St. Louis, MO, USA). Cetyl alcohol (CAl) was supplied by Thermo Scientific (Waltham, MA, USA).

The nomenclature used throughout the manuscript is as follows: BC denotes untreated biochar; CA and NaOH denote acid- and alkaline-treated biochars, respectively; APTS and CAl denote surface functionalization with 3-aminopropyltriethoxysilane and cetyl alcohol, respectively. Combined treatments are designated BC_CA_APTS, BC_CA_CAl, BC_NaOH_APTS and BC_NaOH_CAl. The corresponding vulcanizates are denoted NR_BC, NR_BC_CA, NR_BC_NaOH, etc., while the unfilled reference compound is designated NR0.

The reference formulation contained 100 phr NR, 5 phr ZnO, 2 phr S, 2 phr MBT and 1 phr SA. In the filled formulations, 20 phr of the appropriate BC was additionally introduced.

### 2.2. Preparation of Walnut-Shell Biochar

Approximately 25 g of ground walnut shells were placed in a laboratory crucible and subjected to pyrolysis under a continuous nitrogen atmosphere. Nitrogen was supplied at a flow rate of 200 mL min^−1^. The selected pyrolysis conditions (600 °C for 2 h under a nitrogen atmosphere) were based on conditions reported for the preparation of walnut-shell biochar and were selected to obtain a stable carbonaceous material suitable for subsequent surface modification and application as a reinforcing filler in natural rubber [27].

### 2.3. Chemical Modification of Biochar

Chemical modification was performed in two stages. First, acidic and alkaline treatments were carried out using 10 wt.% CA and 10 wt.% NaOH, respectively. For each treatment, 50 g of BC was introduced into 200 mL of the appropriate solution. The suspensions were stirred at 300 rpm and 55 °C for 2 h. After treatment, the biochar was separated from the treatment solution and repeatedly washed with water to remove residual treatment solution and dissolved inorganic species. The washed materials were subsequently dried at 50 °C for 24 h.

Surface functionalization was subsequently performed using APTS or CAl. For APTS functionalization, 14 g of BC was dispersed in 100 mL of an ethanol/water mixture (80:20, *v*/*v*), and 1.5 mL of APTS was added. The reaction was carried out at 70 °C for 2 h with stirring at 150 rpm. These conditions were selected based on literature reports describing silane functionalization of biochar in ethanol/water media at comparable temperatures and reaction times [28]. For CAl functionalization, 14 g of BC and 2.8 g of CAl were treated under the same solvent, temperature, time and stirring conditions. After functionalization, the solvent was evaporated and the resulting materials were dried at 50 °C for 24 h. No additional washing step was applied after APTS or CAl functionalization. Therefore, the presence of physically adsorbed or unreacted modifier cannot be completely excluded.

**Figure 1:**
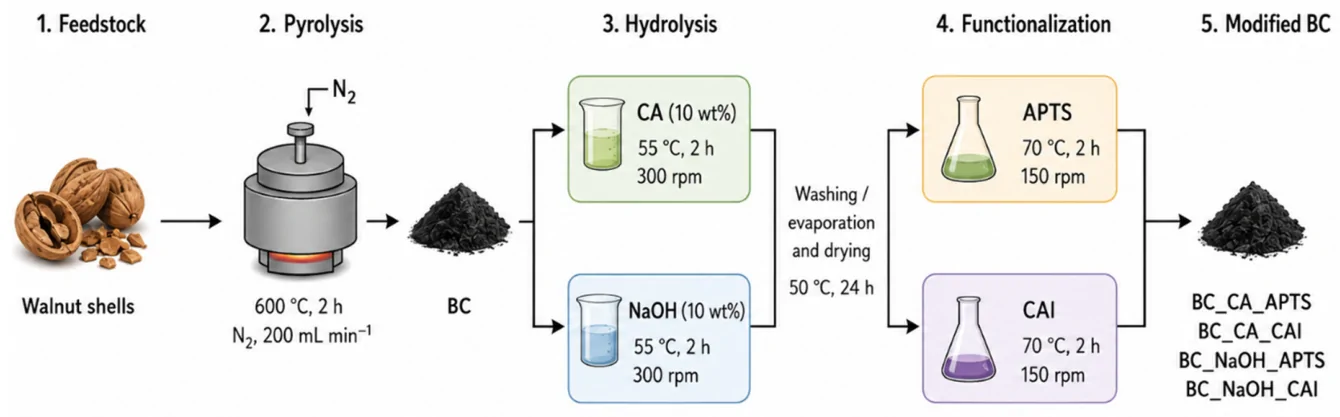
Schematic representation of the preparation and chemical modification of walnut-shell biochar (BC).

A schematic representation of the BC preparation and modification procedure is presented in Figure 1.

### 2.4. Preparation of NR Compounds

The rubber compounds were prepared in two stages. In the first stage, NR was plasticized and mixed with the appropriate BC in a laboratory Brabender internal mixer (Duisburg, Germany) at 50 °C and 50 rpm for approximately 10 min. In the second stage, the premixes were processed on a laboratory two-roll mill with rolls of 450 mm length and 200 mm diameter, a nip gap of 1.5–3 mm, and a front-roll speed of 16 rpm. The vulcanization system was subsequently incorporated.

### 2.5. Vulcanization

Approximately 14 g of each compound was placed in molds and vulcanized at 160 °C for 10 min.

### 2.6. Elemental Analysis

The elemental composition of BC before and after chemical treatment was determined by inductively coupled plasma optical emission spectrometry (ICP-OES) using a PlasmaQuant PQ 9000 Elite (Analytik Jena) (Jena, Germany). The concentrations of Cu, Zn, Mn, Fe, Cr, Ni, Ca, Co and Mg were determined after sample mineralization. Samples were mineralized for 45 min at 240 °C and 60 bar. Calibration was performed using 1000 mg L^−1^ standard solutions (Sigma-Aldrich (St. Louis, MO, USA)).

### 2.7. FTIR Spectroscopy

Fourier transform infrared (FTIR) spectroscopy was used to characterize the chemical structure and surface functional groups of the biochar samples. The analyses were performed using a Nicolet 6700 FTIR spectrometer with OMNIC 3.2 software (Thermo Scientific) (Waltham, MA USA). Spectra were recorded in the range of 400–4000 cm^−1^ using 64 scans at a spectral resolution of 4 cm^−1^.

### 2.8. Thermogravimetric Analysis

The thermal stability of the BC samples was investigated by thermogravimetric analysis (TGA) using a TGA/DSC1 thermal analyzer (Mettler Toledo) (Greifensee, Switzerland). Approximately 10 mg of sample was heated from 25 to 600 °C at 10 °C min^−1^ under an inert atmosphere, followed by oxidation of the residue in air. The temperature corresponding to 10% mass loss (T_10_), the mass loss between 25 and 600 °C (Z_25–600_) and the residual mass after oxidation at 900 °C (R_900_) were determined.

### 2.9. Scanning Electron Microscopy

The surface morphology and particle structure of the biochars were examined using a Quanta 250 FEG environmental scanning electron microscope (ESEM, FEI, Hillsboro, OR, USA) operated in low-vacuum mode at 80 Pa with LFD detection. Micrographs were acquired at different magnifications to characterize the surface morphology and structural features of the biochar particles.

### 2.10. Rheometric Characteristics

The vulcanization behavior of the NR compounds was investigated at 160 °C using a rotorless rheometer D-RPA 3000 (MonTech) (Buchen, Germany). The minimum torque (M_min_), maximum torque (M_max_), torque increment (*Δ*M), scorch time (t_s2_) and optimum cure time (t_90_) were determined from the rheometric curves. The torque increment was calculated according to:

(1) $$\Delta M=M_{max}-M_{min}$$

### 2.11. Differential Scanning Calorimetry

Differential scanning calorimetry (DSC) was performed using a DSC1 instrument (Mettler Toledo) (Greifensee, Switzerland). Samples weighing approximately 8–9 mg were cooled to −100 °C at 10 °C min^−1^ and subsequently heated to 250 °C at the same heating rate under a nitrogen atmosphere. The vulcanization temperature range, apparent heat effect (dCp) and glass transition temperature (Tg) were determined.

### 2.12. Crosslink Density

The crosslink density of the vulcanizates was determined by the equilibrium swelling method in toluene. Samples were immersed in toluene at 25 °C until equilibrium swelling was reached. After swelling, the samples were weighed, dried to constant mass and weighed again. The effective crosslink density ($\nu_{e}$) was calculated using the Flory–Rehner equation [29]:

(2) $$\nu_{e}=-\frac{ln\left(1-V_{r}\right)+V_{r}+\mu V_{r}^{2}}{2V_{0}\left(V_{r}^{1/3}-V_{r}/2\right)}$$

where $\nu_{e}$ is the crosslink density (mol cm^−3^), $V_{0}$ is the molar volume of toluene (106.7 cm^3^ mol^−1^), $V_{r}$ is the volume fraction of the elastomer in the swollen gel, and μ is the Huggins polymer–solvent interaction parameter. For the NR–toluene system at 25 °C, μ was calculated according to:

(3) $$\mu =0.478+0.228V_{r}$$

The volume fraction of elastomer in the swollen gel was calculated from:

(4) $$V_{r}=\frac{m_{s}/\rho_{r}}{m_{s}/\rho_{r}+\left(m_{sw}-m_{s}\right)/\rho_{s}}$$

where $m_{sw}$ is the mass of the swollen sample, $m_{s}$ is the mass of the dry sample, $\rho_{r}$ is the density of NR (0.99 g cm^−3^), and $\rho_{s}$ is the density of toluene (0.86 g cm^−3^).

### 2.13. Static Mechanical Properties

Tensile properties were determined using dumbbell-shaped specimens. The stress at 300% strain (SE_300_), tensile strength (TS) and elongation at break (EB) were determined. The measurements were performed using a Zwick 1435 universal testing machine (Zwick/Roell) (Ulm, Germany).

### 2.14. Hardness

Hardness was determined using the Shore A method. Ten measurements were performed at different positions on each vulcanizate, and the arithmetic mean was used as the final value.

### 2.15. Thermo-Oxidative Aging

Thermo-oxidative aging was performed at 60 °C for 7 days. The aging resistance was evaluated from changes in tensile strength and elongation at break, and expressed using the aging coefficient (K). Crosslink density was additionally determined before and after aging.

The aging coefficient was calculated according to:

(5) $$K=\frac{TS_{a}\;EB_{a}}{TS_{0}\;EB_{0}}$$

where $TS_{0}$ and $EB_{0}$ are the tensile strength and elongation at break before aging, while $TS_{a}$ and $EB_{a}$ are the corresponding values after aging.

### 2.16. Payne Effect

The Payne effect was investigated by dynamic mechanical analysis (DMA) using an ARES-G2 rotational rheometer (TA Instruments) (New Castle, DE, USA) at 25 °C. Circular vulcanizate specimens (25 mm in diameter and 2 mm thick) were tested under a normal force of 2 N at an angular frequency of 10 rad s^−1^. The strain amplitude was progressively increased from 0.1 to 150%. The Payne effect was calculated from the difference between the storage modulus at low and high strain amplitudes:

(6) $$\Delta G^{\prime }=G_{low}^{\prime }-G_{high}^{\prime }$$

where $G_{low}^{\prime }$ and $G_{high}^{\prime }$ represent the storage modulus values determined in the low- and high-strain regions, respectively.

### 2.17. Water Contact Angle

The surface wettability of the vulcanizates was evaluated by the static sessile-drop method. A 10 μL water droplet was deposited on the vulcanizate surface and the contact angle was measured immediately after deposition using an OCA 15EC goniometer (DataPhysics Instruments GmbH) (Filderstadt, Germany).

### 2.18. Statistical Analysis

Mechanical properties and crosslink density were determined using five specimens (*n* = 5), hardness was measured using ten measurements (*n* = 10), and water contact angle measurements were performed using five measurements (*n* = 5). The results are presented as mean ± standard deviation (SD). Statistical analysis was performed using one-way analysis of variance (ANOVA) at a significance level of α = 0.05 (95% confidence level), with *p* < 0.05 considered statistically significant. Rheological measurements, DSC, TGA, Payne effect and FTIR analyses were performed once for each sample (*n* = 1) due to the time-consuming nature of these measurements and the large number of samples investigated. These results are therefore presented as representative measurements and were not subjected to statistical analysis.

## 3. Results and Discussion

### 3.1. Elemental Composition of Biochar and Efficiency of Its Chemical Cleaning

The first stage of biochar modification was aimed at reducing the mineral fraction remaining in the material after biomass pyrolysis. The elemental composition of the walnut shells, untreated biochar (BC), and chemically treated biochars (BC_CA and BC_NaOH) was determined for Cu, Zn, Mn, Fe, Cr, Ni, Ca, Co, and Mg (Table 1). The analyzed elements were present at markedly different concentrations, with Ca, Mg, and Fe representing the most abundant inorganic constituents. Pyrolysis resulted in changes in the concentrations of most analyzed elements, although their magnitude varied depending on the element. The most pronounced increases were observed for Ca, Mg, and Fe, whose concentrations increased from 1.227 to 2.321 wt.%, from 0.276 to 0.516 wt.%, and from 0.153 to 0.233 wt.%, respectively, when comparing the walnut shells with untreated BC. In contrast, the concentrations of Cu and Zn decreased slightly, from 0.007 to 0.002 wt.% and from 0.009 to 0.006 wt.%, respectively. These changes indicate that pyrolysis resulted in a relative enrichment of the solid carbonaceous phase in selected inorganic constituents, primarily Ca, Mg, and Fe, due to the decomposition and removal of a substantial part of the organic fraction.

**Figure 2:**
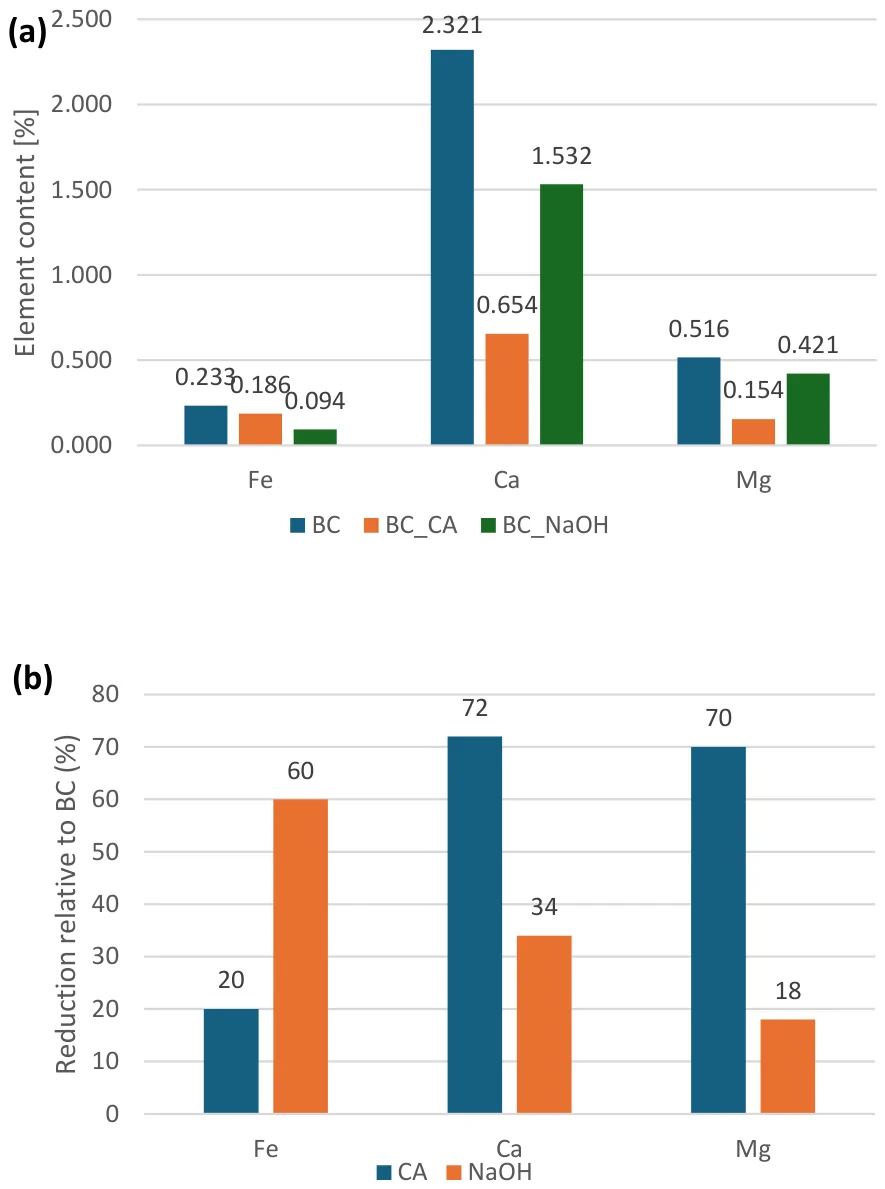
(**a**) Concentrations of Fe, Ca, and Mg in untreated biochar (BC) and after citric acid (BC_CA) or NaOH (BC_NaOH) treatment; (**b**) reduction in Fe, Ca, and Mg content relative to untreated biochar following citric acid and NaOH treatment.

Chemical treatment clearly altered the elemental composition of BC, while the type of treatment determined the effectiveness of removing individual mineral constituents. Citric acid was particularly effective in removing Ca and Mg, whose concentrations decreased from 2.321 to 0.654 wt.% and from 0.516 to 0.154 wt.% (Figure 2a), corresponding to reductions of approximately 72% and 70%, respectively (Figure 2b). In contrast, NaOH was most effective in reducing the Fe content, which decreased from 0.233 to 0.094 wt.% (approximately 60%). The reduction in Ca and Mg following NaOH treatment was considerably lower, amounting to approximately 34% and 18%, respectively.

The observed selectivity of the two treatment methods may result from their different interactions with the mineral fraction of biochar. Citric acid, through acidification and its ability to complex metal ions, can promote the mobilization of Ca^2+^ and Mg^2+^, which may explain their substantially greater removal compared with alkaline treatment [30]. In contrast, the markedly greater reduction in Fe following NaOH treatment indicates that Fe-containing phases present in the biochar were more susceptible to the applied alkaline conditions than to citric-acid treatment. Although ICP-OES does not allow the specific mineralogical forms of the analyzed elements to be identified, the reduction in Fe and other inorganic constituents may be relevant to the subsequent aging behavior of the composites. Metal species have been reported to adversely affect the ageing stability of natural-rubber compounds, a phenomenon commonly referred to as “metal poisoning”. In rubber technology, the term describes the detrimental effect of certain metal ions, particularly transition-metal ions such as Fe, Cu and Mn, on the stability of rubber compounds. These metal species can participate in redox reactions with hydroperoxides formed during rubber oxidation, promoting the formation of reactive species and accelerating oxidative degradation. As a consequence, chain scission and degradation of the crosslinked rubber network may occur, leading to deterioration of mechanical properties during ageing [31,32]. In the present study, the reduction of Fe content from 0.233 wt.% in untreated BC to 0.094 wt.% after NaOH treatment may therefore be relevant to the observed differences in thermo-oxidative ageing behavior. However, the individual contribution of the particular metal species and the detailed mechanism of their interaction with the NR network cannot be established from the present results alone.

### 3.2. Fourier Transform Infrared Spectroscopy (FTIR) Analysis of Biochars

**Figure 3:**
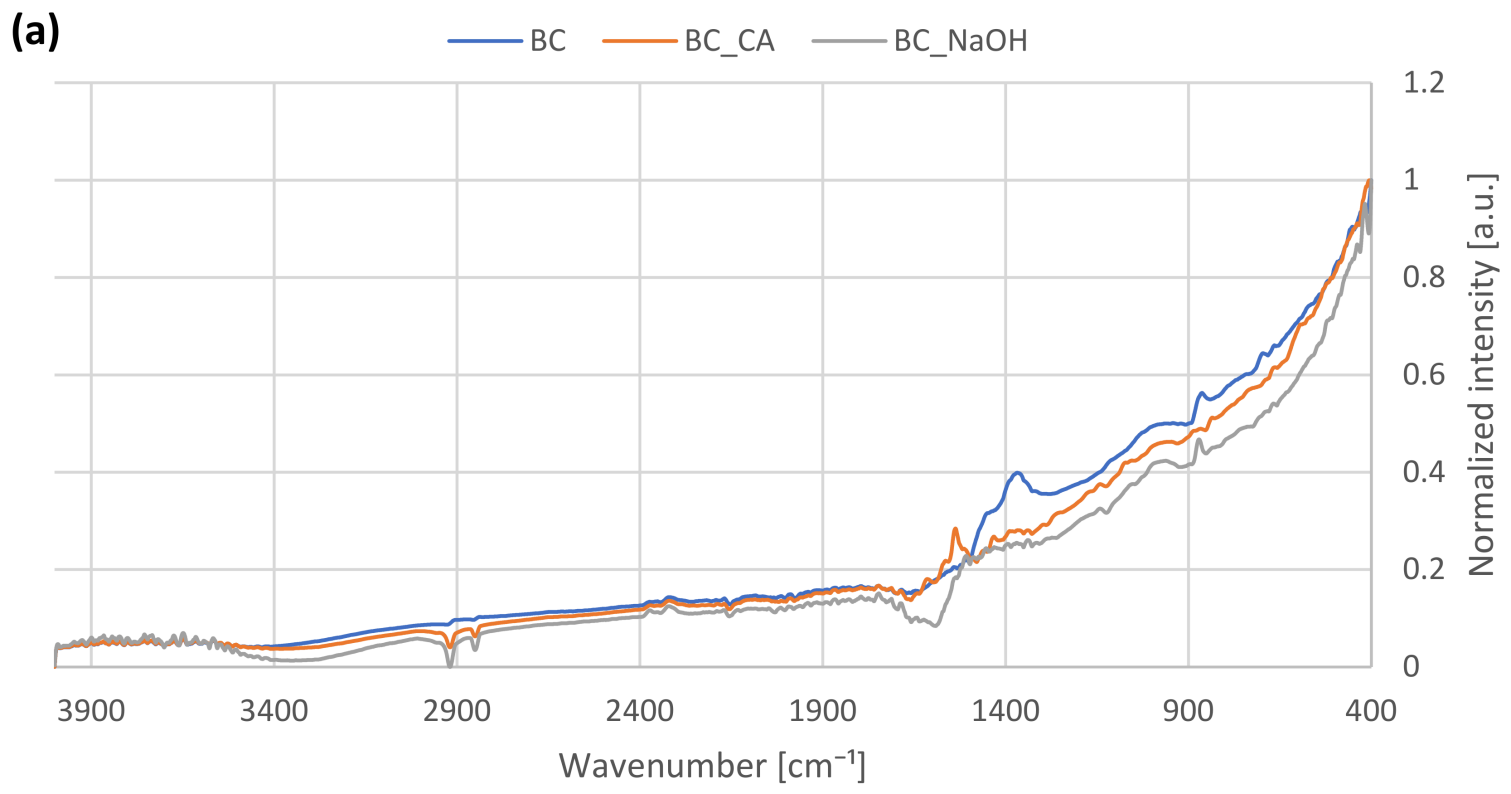

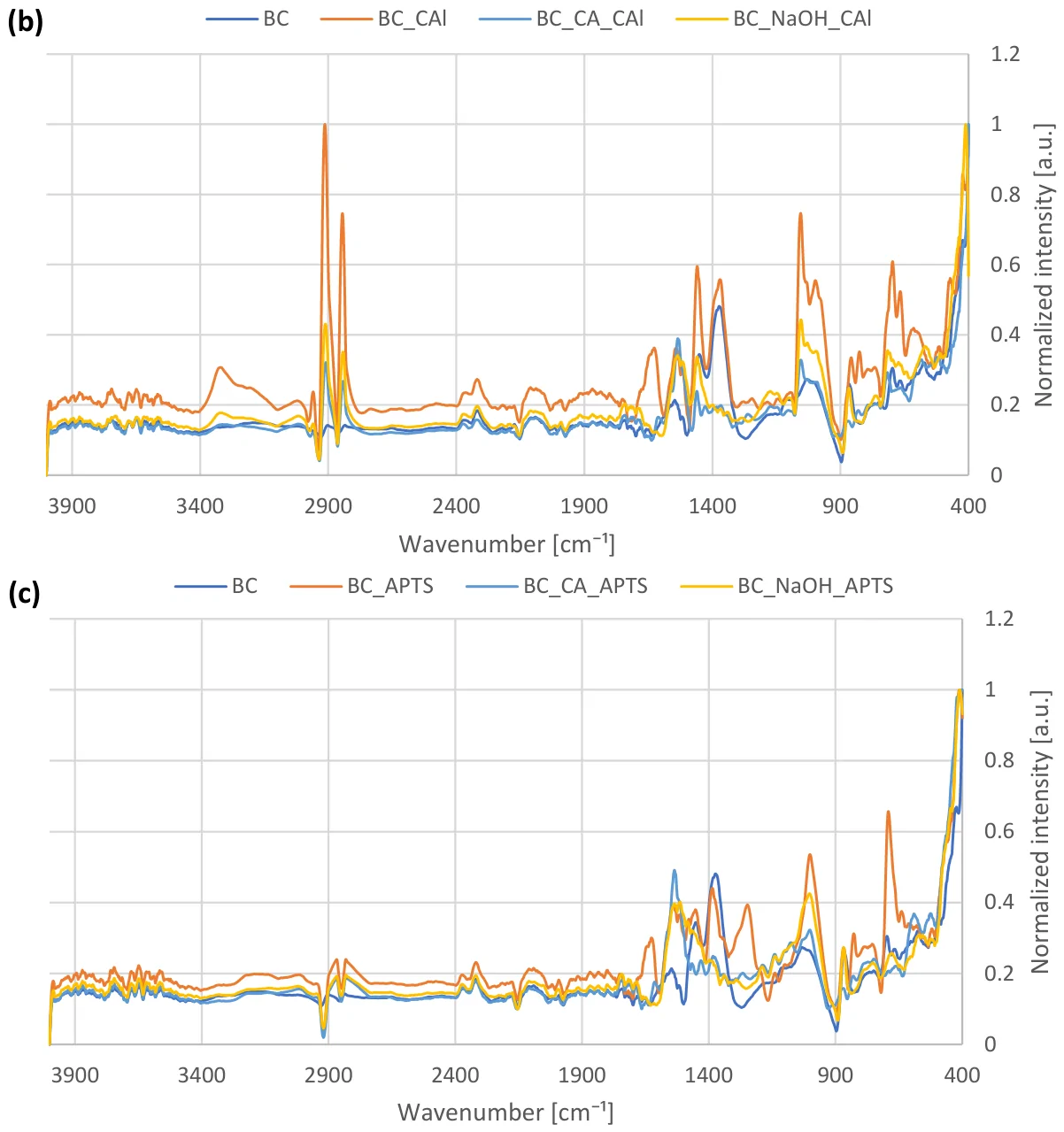
FTIR spectra of walnut-shell biochar after successive chemical cleaning and surface functionalization treatments: (**a**) acid and alkaline modification; (**b**) cetyl alcohol modification; (**c**) APTS modification.

The FTIR spectra presented in Figure 3a revealed characteristic functional groups associated with the carbonaceous structure and residual oxygen-containing functionalities of the investigated biochars. For untreated BC, a broad band in the 3200–3600 cm^−1^ region can be attributed mainly to O–H stretching vibrations of hydroxyl groups, while bands at approximately 2920–2850 cm^−1^ correspond to C–H stretching vibrations of aliphatic groups. The band observed around 1600 cm^−1^ can be associated mainly with aromatic C=C structures and conjugated oxygen-containing groups. In the 1200–1000 cm^−1^ region, the observed signals are primarily related to C–O and C–O–C vibrations. These features are characteristic of lignocellulosic biochars and indicate the coexistence of an aromatic carbonaceous structure with oxygen-containing surface functionalities [33,34,35]. Similar FTIR bands, including O–H stretching at approximately 3300 cm^−1^, aromatic C–H vibrations near 3000 and 870 cm^−1^, and C–O vibrations around 1050 cm^−1^, have been reported for biochars used as elastomeric fillers [36].

The chemical treatment with citric acid and NaOH mainly affected the intensity and relative contribution of the existing absorption bands, particularly those associated with hydroxyl, aliphatic and oxygen-containing groups. At the same time, the characteristic bands associated with the aromatic carbonaceous structure remained present, indicating that the applied cleaning procedures did not substantially disrupt the carbonized framework. The differences between BC_CA and BC_NaOH suggest that the two treatments generated different surface chemical environments, which is consistent with the different elemental-cleaning efficiencies observed by ICP-OES. Thus, the chemical treatment can be considered not only as a method for reducing the mineral fraction but also as a preliminary step controlling the chemical character and availability of surface functionalities for subsequent modification.

More pronounced spectral changes were observed after cetyl alcohol (CAl) functionalization (Figure 3b). In the CAl-containing materials, the bands at approximately 2920 and 2850 cm^−1^ became more pronounced, corresponding to asymmetric and symmetric C–H stretching vibrations of CH_2_ groups, respectively. An increase in the region around 1460 cm^−1^, associated with CH_2_ deformation vibrations, was also observed. The simultaneous occurrence of these bands is characteristic of the long aliphatic chain of cetyl alcohol and provides evidence for its incorporation onto the biochar surface. The corresponding changes were also observed for BC_CA_CAl and BC_NaOH_CAl, although their intensity differed depending on the preceding chemical treatment. This suggests that removal of the mineral fraction and modification of the surface chemistry affected the interaction of CAl with BC. Nevertheless, the FTIR results alone do not allow the interaction to be unequivocally classified as covalent; adsorption or surface association of the organic modifier may also contribute to the observed spectral changes.

The spectra of APTS-functionalized biochars (Figure 3c) exhibited more complex changes, particularly in the 2920–2850, 1650–1500, and 1200–900 cm^−1^ regions. The bands around 2920–2850 cm^−1^ can be associated with C–H vibrations of the propyl groups introduced with APTS, whereas changes in the 1650–1500 cm^−1^ region may include contributions from vibrations associated with nitrogen-containing functionalities. Particular attention should be paid to the 1200–900 cm^−1^ region, where the native C–O/C–O–C vibrations of BC overlap with vibrations that may originate from Si–O–C and Si–O–Si groups formed during silane hydrolysis and condensation. Differences between the samples are also visible in the low-wavenumber region below 900 cm^−1^ [37]. Although this region cannot be assigned exclusively to silane-derived structures because of overlapping contributions from the carbonaceous and mineral fractions, the changes observed after APTS treatment support the incorporation of silicon-containing functionalities onto the BC surface. Similar changes in the 1000–1100 and below-900 cm^−1^ regions have been reported for silanized biochar and other lignocellulosic substrates modified with organosilanes [37,38].

### 3.3. Thermal Stability of Biochars

The thermal stability of the investigated biochars differed substantially depending on the applied modification (Table 2). The analysis was conducted from 25 to 600 °C under an argon atmosphere, followed by heating from 600 to 900 °C in air. Untreated BC exhibited the highest T_10_ (407 °C), indicating high thermal stability of its carbonaceous structure. Considerably lower T_10_ values were obtained for BC_APTS and BC_CAl, at 165 and 179 °C, respectively. This decrease does not necessarily indicate reduced stability of the carbonaceous phase itself, but may result from the presence of additional, thermally less stable organic species introduced during surface functionalization.

This interpretation is supported by the Z_25–600_ values, which increased from 13.9% for BC to 21.4% for BC_APTS and 28.0% for BC_CAl. The particularly high mass loss observed for the CAl-functionalized materials indicates a greater contribution of an organic fraction decomposing within this temperature range. Chemically treated biochars exhibited intermediate T10 values: 363 °C for BC_CA and 267 °C for BC_NaOH, while subsequent APTS functionalization resulted in only minor changes, to 355 and 266 °C, respectively. In contrast, the CAl-functionalized materials exhibited T_10_ values of 179–189 °C, confirming the pronounced contribution of the introduced organic phase to the initial stage of thermal decomposition.

The R_900_ values also changed markedly following chemical treatment. R_900_ decreased from 31.6% for BC to 22.3% for BC_CA and approximately 16% for the NaOH-treated materials. Since the 600–900 °C stage was conducted in air, the lower residue indicates a reduction in the non-oxidizable fraction following chemical treatment. This result is consistent with the ICP-OES analysis and confirms the effectiveness of the applied treatments in reducing the mineral content of the biochars. In particular, the pronounced decrease in R_900_ after NaOH treatment corresponds well with the previously observed reduction in Fe, Ca, and Mg content.

### 3.4. Morphology of Walnut-Shell Biochars

The SEM analysis was primarily used to evaluate the morphological changes in walnut-shell biochar resulting from the applied chemical purification treatments. Therefore, the analysis focused on untreated BC and BC treated with CA or NaOH. The effects of subsequent APTS and CAl functionalization were evaluated primarily via FTIR spectroscopy and TGA. Since SEM measurements were performed using external analytical facilities, the number of analyses was limited; therefore, SEM characterization was focused on the samples most relevant for assessing the morphological effects of the chemical purification step.

**Figure 4:**
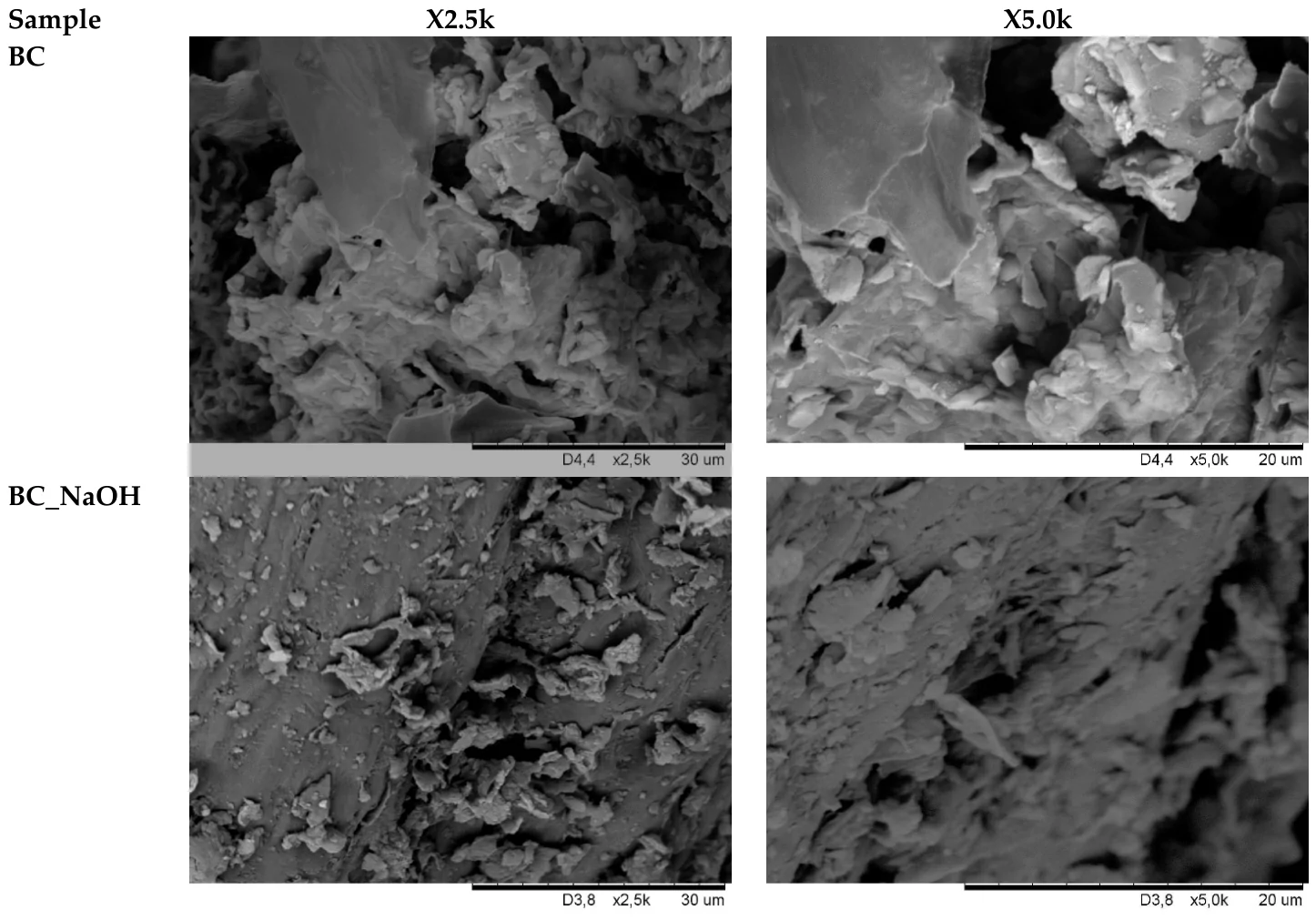

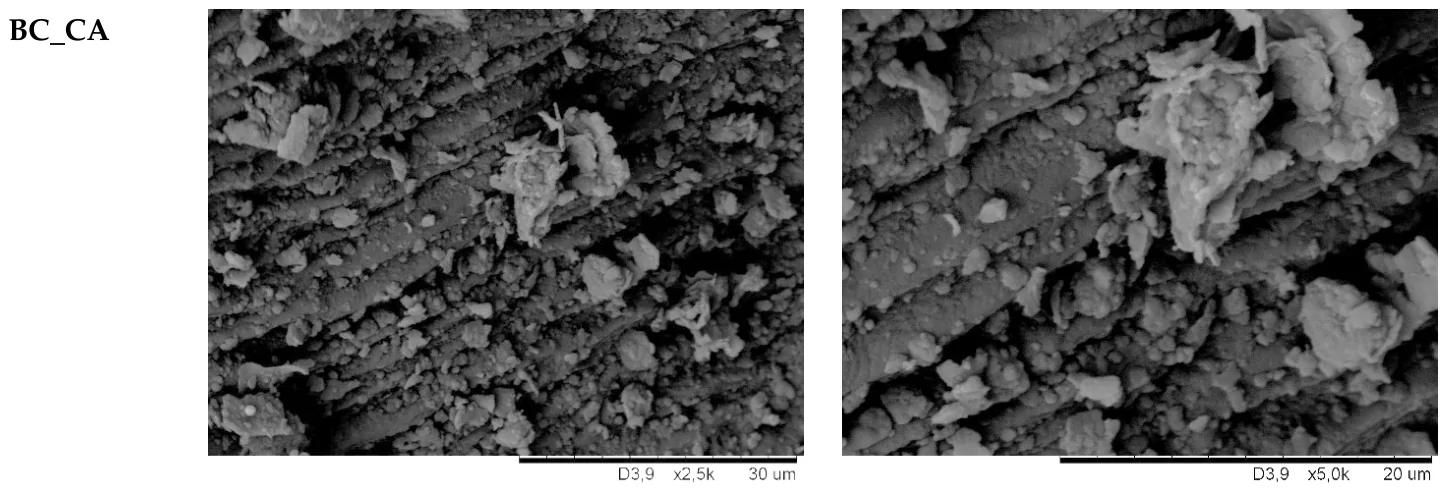
SEM micrographs of walnut-shell biochar at selected magnifications: untreated BC, BC_NaOH and BC_CA.

The SEM images presented in Figure 4 reveal a clearly heterogeneous morphology of the walnut-shell biochar, characterized by irregular particle shapes, rough surfaces, and the presence of cracks, cavities, and pores. The developed porous structure is typical of biochars obtained from lignocellulosic feedstocks. Such morphology increases the effective contact area with the polymer matrix and may promote mechanical anchoring of the rubber within surface irregularities and pores of the filler.

The applied chemical modifications resulted in visible changes in the surface morphology, particularly in the number and character of cavities and surface irregularities. The observed changes may be associated with the partial removal of the mineral fraction, in agreement with the ICP-OES results, leading to the exposure of additional regions of the carbonaceous structure. Overall, the SEM observations indicate that the applied treatments modified not only the chemical composition but also the surface morphology of the biochar, potentially affecting its subsequent interactions with the NR matrix.

### 3.5. Rheometric Behavior and Vulcanization Kinetics of Rubber Mixtures

**Figure 5:**
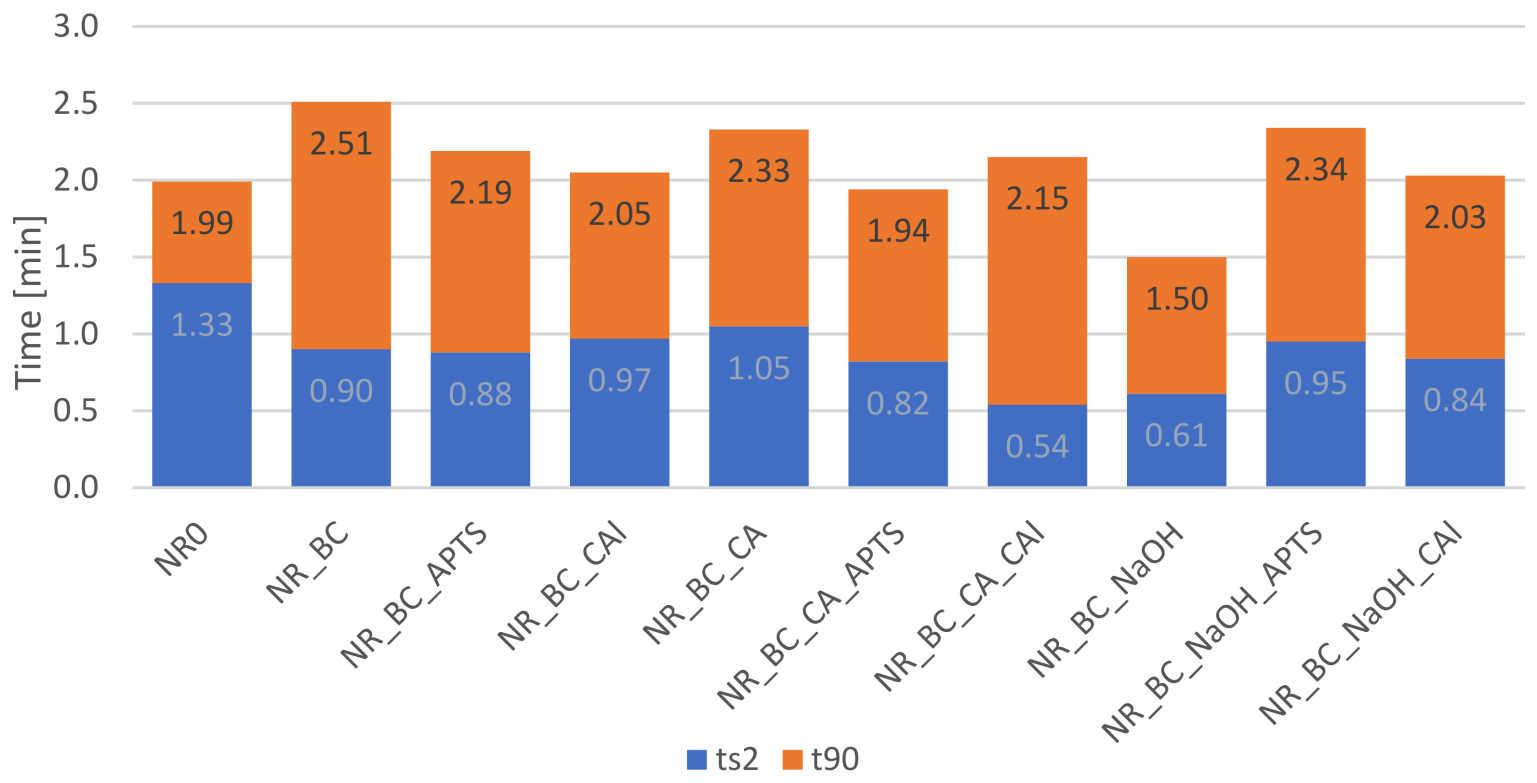
Scorch time (t_s2_) and optimum cure time (t_90_) of the investigated natural rubber compounds.

The rheometric characteristics are presented in Table 3, while the scorch and optimum cure times are shown in Figure 5. The incorporation of BC increased ΔM from 2.88 dNm for NR0 to 4.39 dNm for NR_BC, indicating a more developed vulcanized structure. The highest *Δ*M (4.55 dNm) and M_max_ (4.89 dNm) were obtained for NR_BC_NaOH. This indicates a particularly favorable effect of alkaline BC treatment on the development of the vulcanized structure, likely associated with both the presence of the rigid filler phase and its interactions with the NR matrix [39,40]. However, *Δ*M should not be considered solely as a direct measure of crosslink density since it is also affected by filler–filler and filler–rubber interactions [6].

The effect of surface functionalization was particularly evident in M_min_. CAl-containing systems generally exhibited lower M_min_ values than their corresponding non-functionalized materials. This may indicate easier flow of the compound and reduced filler–filler interactions associated with the presence of long, non-polar alkyl chains [6]. In contrast, APTS resulted in a slight increase in M_min_, particularly for NR_BC_NaOH_APTS. At the same time, both CAl and APTS reduced M_max_ compared with the corresponding non-functionalized materials, suggesting that, in the present system, the chemical cleaning step had a stronger effect on the development of the vulcanized structure than subsequent surface functionalization.

Changes in t_s2_ and t_90_ (Figure 5) further confirmed the influence of BC modification on vulcanization kinetics. For NR0, these values were 1.33 and 1.99 min, respectively, whereas the incorporation of untreated BC increased t_90_ to 2.51 min. The strongest acceleration of the process was observed for NR_BC_NaOH, for which t_s2_ = 0.61 min and t_90_ = 1.50 min. The combination of the shortest curing times with the highest *Δ*M indicates a particularly favorable effect of alkaline BC treatment on the vulcanization process, consistent with previous reports on the influence of biochar surface characteristics on NR curing behavior [41]. CAl primarily reduced t_s2_, with the lowest value (0.54 min) obtained for NR_BC_CA_CAl, but this was not accompanied by a proportional reduction in t_90_. This suggests that CAl may predominantly affect the initial stage of vulcanization, whereas the effect of NaOH extends throughout the curing process.

The shorter optimum cure time observed for the NR_BC_NaOH compound (t90 = 1.50 min) compared with NR_BC (2.51 min) may be associated with changes in the surface chemistry and mineral composition of BC induced by alkaline treatment. The possible contribution of residual alkaline species should also be considered; however, the pH and residual NaOH content were not determined in the present study, and therefore this mechanism cannot be confirmed directly. In sulfur-vulcanized rubber systems, zinc oxide and stearic acid are commonly used as inorganic and organic activators, respectively, and their interaction with organic accelerators plays an important role in determining the efficiency and rate of vulcanization [42]. The surface chemistry of fillers can also substantially affect the curing process. For example, the acidic surface of silica may interact with components of the vulcanization system and retard the cure reaction by reducing the availability of active zinc species and/or accelerator [42]. Thus, the faster curing observed for the NaOH-treated BC may result from the combined effect of its modified surface chemistry, inorganic composition and possible alkaline character. However, because residual NaOH was not quantified, its direct contribution to the observed cure acceleration cannot be established from the present results alone.

From the perspective of conventional rubber compounding, carbon black and silica are among the most widely used reinforcing fillers in rubber technology, and their surface characteristics strongly influence both the vulcanization behavior and the resulting properties of rubber compounds [43]. In particular, silica contains surface silanol groups that can interact with zinc-containing components of the curing system, whereas carbon black generally exhibits a different surface chemistry and interaction with rubber and compounding ingredients [44]. The present results therefore indicate that, similarly to conventional fillers, modification of the BC surface can substantially affect its interaction with the NR vulcanization system. However, the present study was not designed to establish a direct equivalence between walnut-shell BC and conventional reinforcing fillers.

### 3.6. Differential Scanning Calorimetry (DSC) Analysis of Vulcanization and Glass Transition of Rubber Compounds

The rheometric analysis was complemented by differential scanning calorimetry (DSC) to evaluate the temperature range associated with vulcanization and changes in the glass transition temperature of NR. The results are presented in Table 4. For the unfilled NR0 compound, the vulcanization range was 158–211 °C, whereas the incorporation of untreated BC narrowed this range to 157–189 °C. This is consistent with the increase in t_90_ observed by rheometry for NR_BC, indicating a different course of the crosslinking process in the presence of untreated biochar.

The applied surface cleaning and functionalization resulted in both narrowing and broadening of the vulcanization temperature range. Particularly broad ranges were observed for NR_BC_CA (155–218 °C) and NR_BC_CA_APTS (121–209 °C). In contrast, the range for NR_BC_NaOH was 142–214 °C, which corresponds well with the rheometric reduction in both t_s2_ and t_90_. These differences indicate that the method of BC surface modification affects not only the vulcanization rate but also the temperature range over which processes associated with network formation occur.

Pronounced differences between the formulations were also observed for dCp. The highest value was obtained for NR_BC_CA_APTS (23.69 J/g), whereas NR_BC_APTS and NR_BC_CAl exhibited considerably lower values of 4.44 and 4.18 J/g, respectively. The absence of a clear relationship between dCp and the type of modification indicates that the thermal effect observed during DSC results from the combined influence of the compound composition and the surface characteristics of BC.

In contrast to the vulcanization range and dCp, Tg remained within a relatively narrow range of −65.34 to −62.65 °C. These small changes indicate that BC modification had a limited effect on the segmental mobility of NR chains. The observed shifts in Tg may, however, indicate changes in the local polymer environment resulting from the presence and modification of the filler surface.

### 3.7. Crosslink Density of Rubber Compounds by Equilibrium Swelling

The calculated crosslink density values are presented in Table 5. Compared with the unfilled NR0 compound (2.35 × 10^−5^ mol/cm^3^), the incorporation of untreated BC increased the crosslink density to 2.84 × 10^−5^ mol/cm^3^, indicating that the presence of BC favored the development of the three-dimensional rubber network. A similar increase in crosslink density has been reported for NR containing walnut shells, confirming that biofillers can influence the efficiency of sulfur vulcanization [9].

The most pronounced effect was observed for APTS-functionalized BC. The crosslink density increased to 3.45 × 10^−5^ mol/cm^3^ for NR_BC_APTS, while similarly high values were obtained for NR_BC_NaOH_APTS (3.23 × 10^−5^ mol/cm^3^) and NR_BC_CA_APTS (3.04 × 10^−5^ mol/cm^3^). Thus, regardless of the preceding chemical treatment, APTS consistently promoted the formation of a denser rubber network. This agrees well with previous studies of NR/biofiller composites, in which APTS increased crosslink density and improved the curing behavior of systems containing walnut shells and starch [9].

The beneficial effect of APTS can be related to the aminopropyl groups introduced onto the filler surface. These functional groups can modify the local chemical environment at the filler–rubber interface and interact with components of the vulcanization system, thereby facilitating network formation [45,46]. Previous studies have shown that APTS can enhance the curing process, filler dispersion and rubber–filler interactions in natural rubber composites [47]. In silica-filled NR, APTS was reported to promote curing and increase rubber–filler interactions, while studies on biofillers have attributed the increase in crosslink density to improved filler dispersion and more efficient vulcanization [9,48].

The effect of CAl was considerably less pronounced. NR_BC_CAl showed a crosslink density (2.83 × 10^−5^ mol/cm^3^) similar to that of untreated BC, whereas the combination of citric-acid treatment and CAl resulted in the lowest value (2.11 × 10^−5^ mol/cm^3^). In contrast, CAl applied after NaOH treatment resulted in 3.00 × 10^−5^ mol/cm^3^. This variation indicates that the effect of surface functionalization depended strongly on the chemical state of the BC surface established during the preceding cleaning step.

Overall, the results demonstrate that APTS was the most effective modification for increasing the crosslink density of NR/BC vulcanizates, whereas the effect of CAl was more dependent on the preceding chemical treatment. The higher crosslink density of the APTS-containing systems is also consistent with the rheometric results, particularly the changes in *Δ*M and curing characteristics, supporting the conclusion that aminosilane functionalization promoted the development of the NR crosslinked network.

It should also be noted that natural rubber contains non-rubber constituents, including proteins and fatty acid derivatives, which may interact with polyisoprene chains, filler surfaces and components of the vulcanization system. These constituents may therefore contribute to the observed differences in crosslinking behavior. However, their individual contribution was not investigated in the present study and cannot be separated from the effects associated with BC modification.

### 3.8. Mechanical Properties and Hardness of Vulcanized Rubber Composites

**Figure 6:**
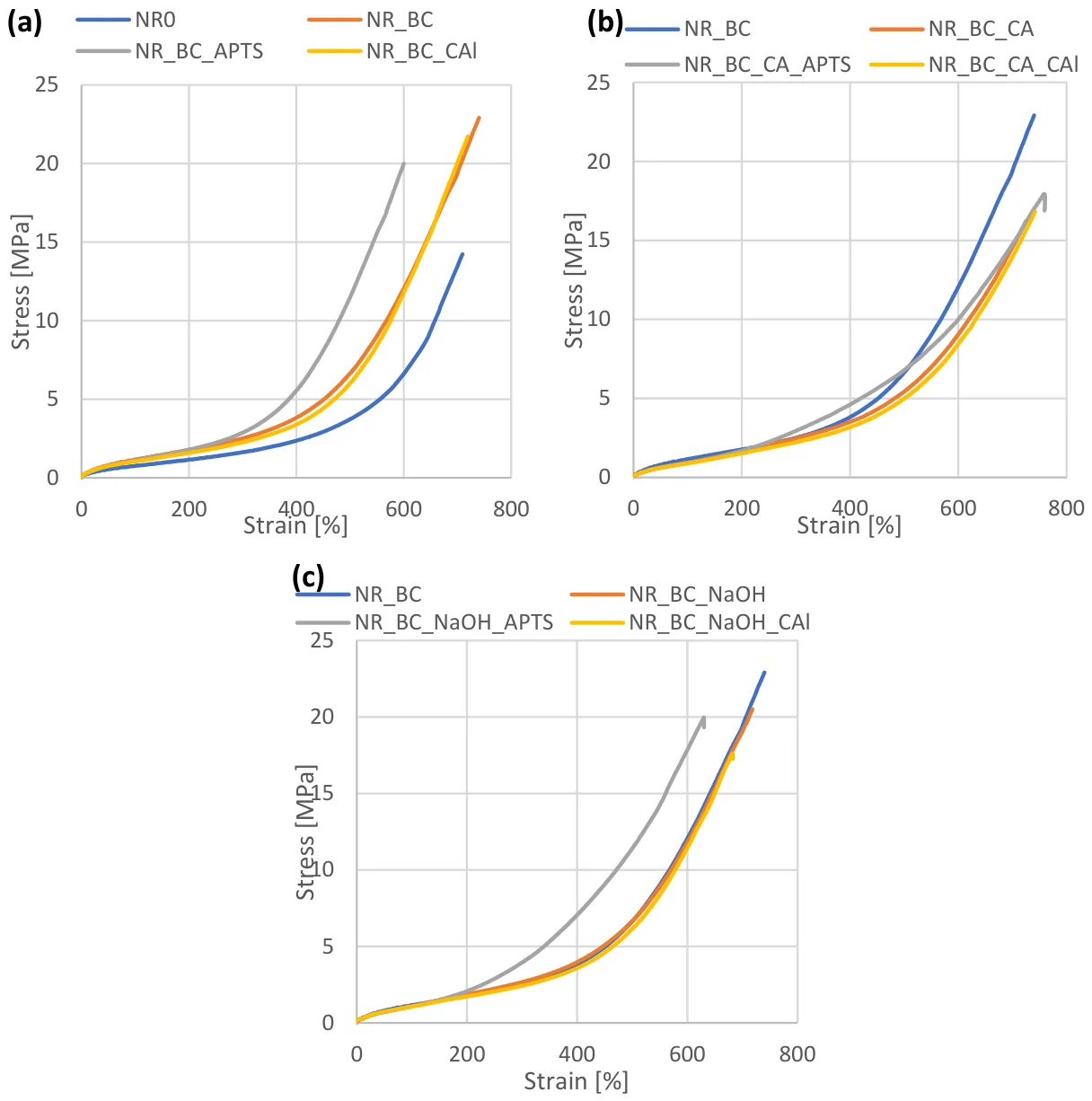
Stress–strain curves of the investigated natural rubber vulcanizates: (**a**) untreated and functionalized biochar, (**b**) citric-acid-treated biochar and its functionalized derivatives, and (**c**) NaOH-treated biochar and its functionalized derivatives.

**Figure 7:**
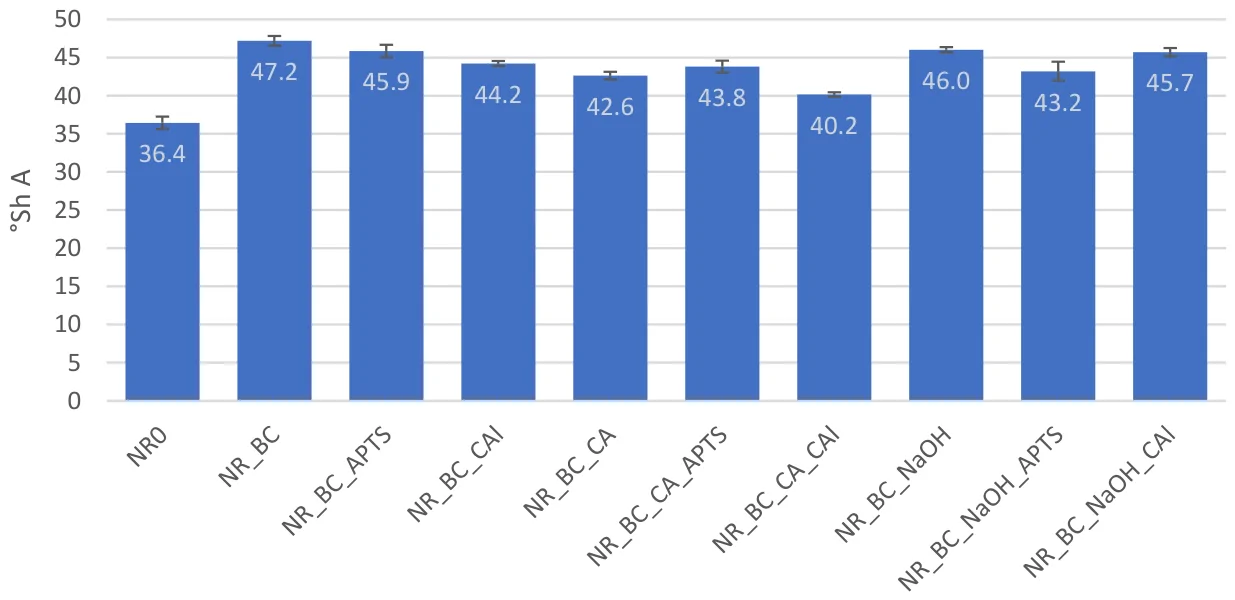
Hardness (Shore A) of the investigated natural rubber vulcanizates.

The mechanical properties and hardness of the NR vulcanizates are presented in Figure 6 and Figure 7 and Table 6. Incorporation of untreated BC resulted in a clear improvement in the mechanical performance compared with NR0. The SE_300_ increased from 1.79 to 2.47 MPa, while TS increased from 14.1 to 22.1 MPa, with a simultaneous slight increase in EB from 708 to 734%. The stress–strain curve of NR_BC also shows higher stress levels at large strains compared with the unfilled NR, confirming the reinforcing effect of BC. Similar increases in stiffness and tensile properties following the incorporation of biochar into NR have been reported in recent studies [39,41].

The effect of subsequent BC functionalization was more differentiated. The APTS-containing vulcanizates exhibited the highest SE_300_ values, reaching 3.44 MPa for NR_BC_APTS, 3.20 MPa for NR_BC_CA_APTS and 3.92 MPa for NR_BC_NaOH_APTS. This behavior is also evident from the stress–strain curves (Figure 6), where APTS-functionalized systems generally show a steeper increase in stress with increasing strain, particularly in the high-strain region. This indicates a more resistant and stiffer network, consistent with the increased crosslink densities determined for these materials. The beneficial effect of APTS on the crosslinking and mechanical behavior of NR composites containing biofillers has also been reported previously, including systems based on walnut shells [9].

The increase in stiffness induced by APTS was accompanied by a reduction in EB. For NR_BC_APTS, EB decreased from 734 to 600%, while for NR_BC_NaOH_APTS it decreased from 713 to 633%. At the same time, TS remained relatively high, at 20.1 and 20.0 MPa, respectively. This combination indicates that APTS modification shifted the mechanical response towards greater resistance to deformation rather than maximizing ultimate extensibility. Such behavior is consistent with the effect of increased crosslink density on the tensile response of NR vulcanizates.

In contrast, the effect of CAl functionalization was less uniform and depended strongly on the preceding BC treatment. NR_BC_CAl retained relatively high mechanical performance, with SE_300_ = 2.24 MPa, TS = 21.6 MPa and EB = 717%. Following citric-acid treatment, CAl resulted in the highest elongation at break among the investigated materials (753%), accompanied by lower SE_300_ (2.18 MPa) and TS (17.1 MPa). This behavior corresponds to the relatively low crosslink density of NR_BC_CA_CAl and indicates a less stiff network. After NaOH treatment, the corresponding values were 2.42 MPa, 18.7 MPa and 717%, respectively, confirming that the effect of functionalization was strongly dependent on the chemical state of the BC surface.

The hardness results (Figure 7) provide additional evidence of the stiffening effect of BC. The incorporation of untreated BC increased hardness from 36.4 Shore A for NR0 to 47.2 Shore A for NR_BC, representing the highest value among all investigated formulations. After functionalization, hardness remained relatively high, ranging from 40.2 to 45.9 Shore A. The highest values were obtained for NR_BC_APTS (45.9 Shore A) and NR_BC_NaOH (46.0 Shore A), whereas the lowest value was observed for NR_BC_CA_CAl (40.2 Shore A). The increase in hardness after biochar incorporation is consistent with the formation of a more rigid composite structure and has also been reported for other NR/biochar systems [49].

Overall, the mechanical and hardness results demonstrate that BC effectively reinforces the NR matrix, while APTS modification most consistently increases network stiffness, as reflected by the higher SE_300_ and steeper stress–strain response; in contrast, the effect of CAl depends strongly on the preceding chemical treatment of BC.

### 3.9. Aging Resistance of Vulcanized Rubber Composites

**Figure 8:**
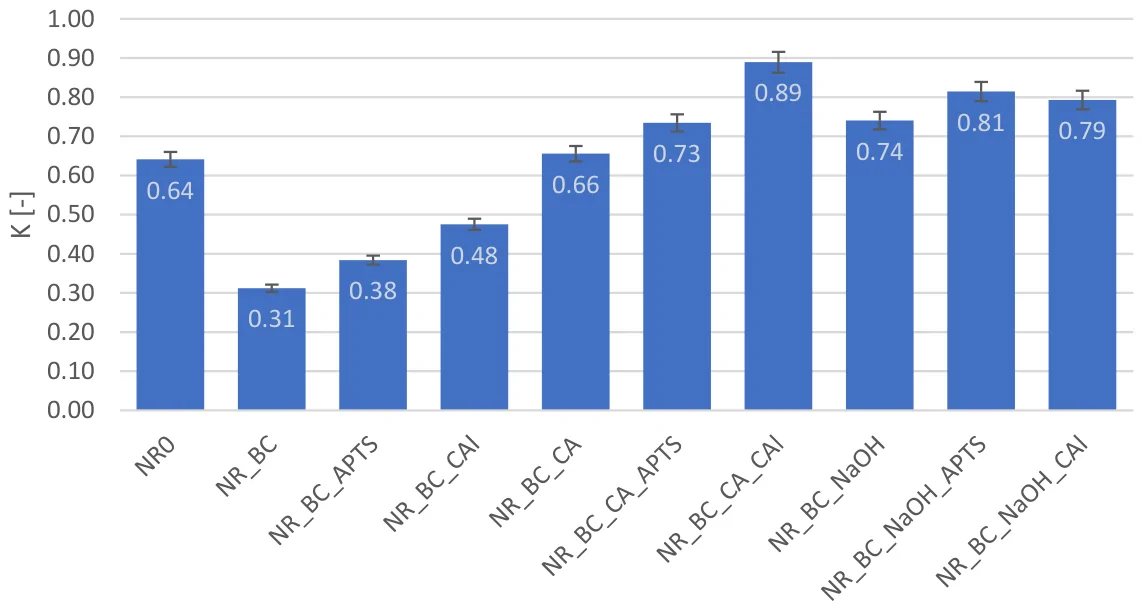
Aging coefficient (K) of the NR vulcanizates after thermo-oxidative aging.

**Figure 9:**
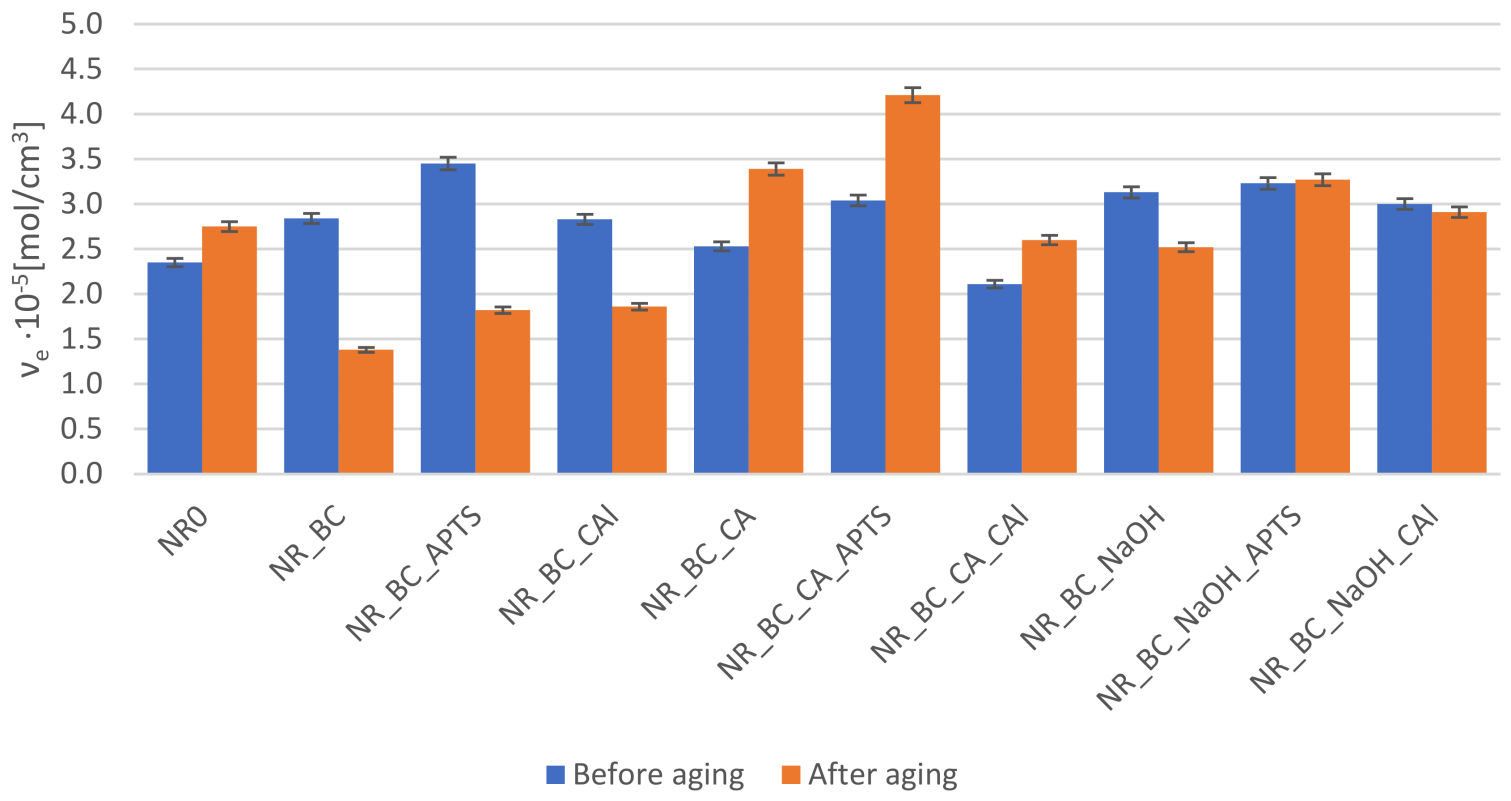
Changes in crosslink density (ν_e_) of the NR vulcanizates before and after thermo-oxidative aging.

The aging resistance of the NR vulcanizates was evaluated based on the aging coefficient K and changes in crosslink density ν_e_ before and after thermo-oxidative aging (Figure 8 and Figure 9). NR0 exhibited a K value of 0.64, whereas incorporation of untreated BC reduced this value to 0.31, indicating a pronounced deterioration in aging resistance. Surface functionalization of untreated BC partially improved this behavior, increasing K to 0.38–0.48. A much stronger improvement was observed after chemical purification of BC. The K values reached 0.66–0.89 for citric-acid-treated systems and 0.74–0.81 for NaOH-treated systems, with the highest value obtained for NR_BC_CA_CAl (K = 0.89).

The improved aging resistance after BC purification can be related to changes in its inorganic composition. Elemental analysis showed a reduction in Fe, Ca and Mg content after chemical treatment. This is particularly relevant because transition-metal ions, including Fe, Mn and Cu, can catalyze rubber oxidation through reactions involving hydroperoxides [13]. Their partial removal may therefore reduce the oxidative activity of BC. The changes in surface chemistry identified by FTIR may additionally contribute to the different behavior of the modified fillers.

Changes in ν_e_ further confirmed the different responses of the vulcanizates to aging. NR0 showed an increase in ν_e_ from 2.35 to approximately 2.75 × 10^−5^ mol/cm^3^, indicating further development of the crosslinked structure during aging. In contrast, untreated BC caused a pronounced decrease in ν_e_, from 2.84 to approximately 1.40 × 10^−5^ mol/cm^3^, which is consistent with a greater extent of network degradation. Functionalization of untreated BC with APTS or CAl did not prevent this decrease. A different behavior was observed for chemically purified BC, particularly after citric-acid treatment, where ν_e_ generally increased after aging. For NaOH-treated systems, the changes were smaller, with the APTS-functionalized material showing nearly unchanged ν_e_. These differences reflect the balance between additional crosslinking and degradation of polymer chains and crosslinks during thermo-oxidative aging [50].

The non-rubber constituents naturally present in NR, including proteins and fatty acid derivatives, may also contribute to this behavior through their interactions with polyisoprene chains, filler surfaces and the vulcanization network. Their potential contribution to the aging response cannot, however, be distinguished from the effects of BC purification and functionalization based on the present results.

The comparison of functionalization routes also revealed a selective effect of APTS and CAl depending on the purification method. CAl was most effective after citric-acid treatment, giving the highest K value (0.89), whereas APTS provided the highest K after NaOH treatment (0.81). This suggests that the aging performance resulted from the combined effect of BC purification and surface functionalization rather than from functionalization alone.

### 3.10. Analysis of the Payne Effect in Biochar-Filled Vulcanized Rubber Composites

**Figure 10:**
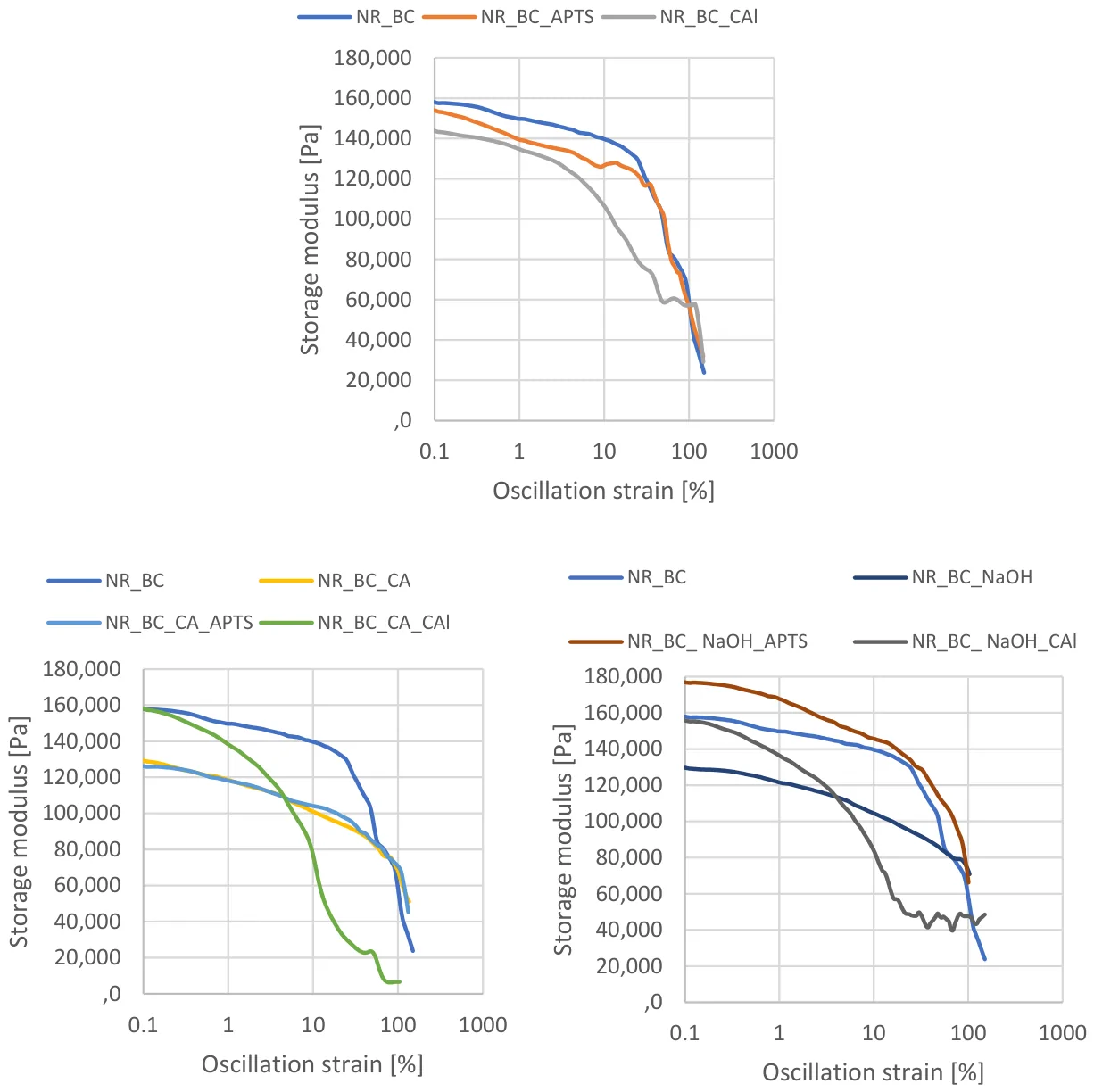
Storage modulus (G′) as a function of oscillation strain amplitude for the investigated NR/BC compounds.

The Payne effect was evaluated from the decrease in storage modulus (G′) with increasing oscillation strain amplitude (Figure 10). All investigated compounds exhibited a characteristic decrease in G′ with increasing strain, particularly in the low-strain region. This behavior is typical of filled rubber systems and reflects the progressive breakdown of the filler–filler network under deformation [51]. The magnitude of this decrease varied considerably between the formulations, which was reflected in the calculated Payne effect values.

The unmodified BC system exhibited a relatively high Payne effect of 75.5%, indicating pronounced strain-dependent softening and strong filler–filler interactions. Surface functionalization of untreated BC slightly reduced this value to 70.9% for APTS and 64.8% for CAl, suggesting a weakening of the filler network (Table 7). A more pronounced reduction was observed after citric-acid treatment, for which the Payne effect decreased to 51.8% and further to 49.0% after APTS functionalization. In contrast, CAl functionalization after CA treatment resulted in a very high Payne effect of 95.8%, accompanied by a particularly pronounced decrease in G′ with increasing strain, indicating strong filler–filler interactions.

The NaOH-treated systems showed a different response. The lowest Payne effect was obtained for NR_BC_NaOH (40.1%), indicating relatively weak filler–filler interactions. APTS increased the value to 49.5% (Table 7), whereas CAl resulted in a substantially higher value of 70.3%. Thus, the influence of functionalization was clearly dependent on the preceding chemical treatment.

The differences in the shape of the G′–strain curves and in the calculated Payne effect demonstrate that the applied purification and functionalization procedures substantially modified the strength of the filler network. In particular, the lower Payne effect observed for CA- and NaOH-treated systems indicates reduced filler–filler association, whereas the exceptionally high value for CA_CAl suggests the formation of a considerably stronger filler network [52,53].

### 3.11. Wettability and Contact Angle of Biochar-Filled Rubber Composites

**Figure 11:**
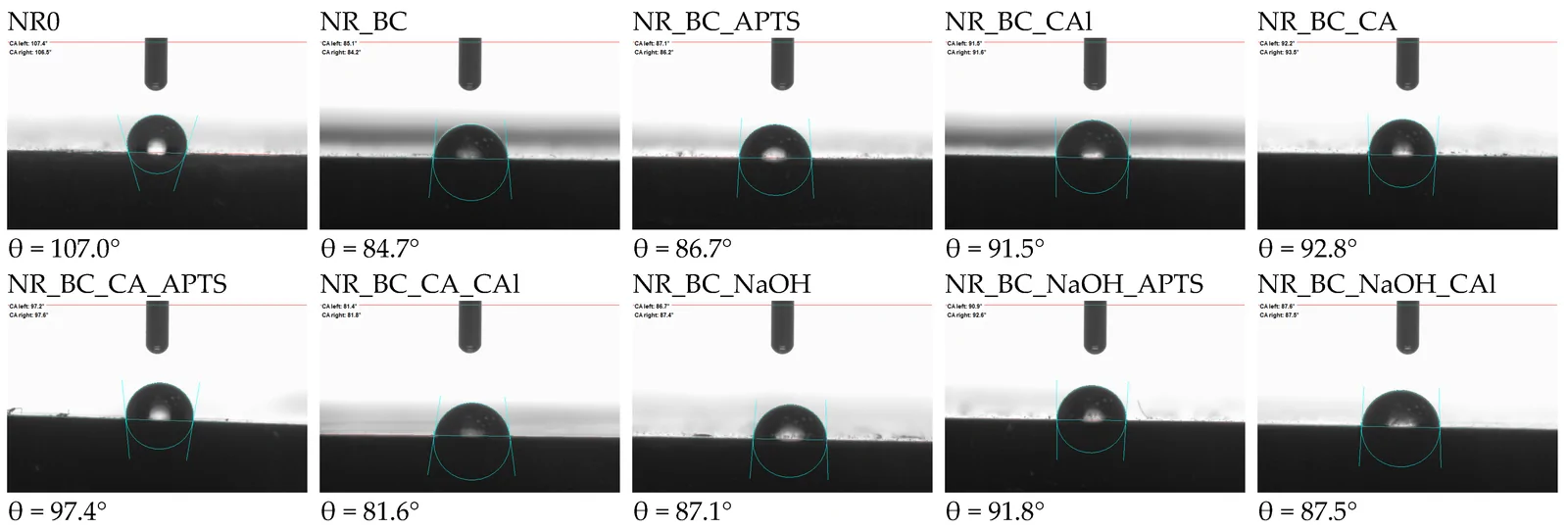
Water contact angle of the investigated NR/BC systems.

The water contact angle measurements further confirmed that the applied treatments affected the surface characteristics of the investigated NR/BC vulcanizates (Figure 11). The reference NR0 exhibited the highest contact angle (107.0°), whereas incorporation of untreated BC reduced this value to 84.7°, indicating an increase in the wettability of the vulxcanizate surface. This change may be associated with the presence of polar oxygen-containing groups introduced with BC, as indicated by the FTIR analysis. However, since the measurements were performed on the vulcanizate surface, the measured contact angle reflects the combined surface characteristics of the NR matrix and BC, including the composition, distribution and surface exposure of the filler, rather than the intrinsic wettability of the BC particles.

Surface modification of BC resulted in further changes in the measured contact angle. depending on the applied treatment. For APTS-functionalized BC. the contact angle increased to 86.7°, whereas CAl modification resulted in 91.5°. A greater variation was observed after citric-acid treatment: the contact angle was 92.8° for BC_CA, increased to 97.4° after APTS functionalization, while CAl modification reduced it to 81.6°. Following NaOH treatment, the contact angles remained within a relatively narrow range of 87.1–91.8°, with the highest value obtained for the APTS-functionalized material. These differences indicate that the effect of surface functionalization on the measured surface wettability of the NR/BC vulcanizates depended strongly on the chemical state of BC generated during the preceding purification step. Similar changes in surface wettability have been reported following silane modification, demonstrating that surface functionalization can substantially alter the balance between polar and non-polar surface interactions [28].

It should be emphasized that the measured contact angles characterize the surface wettability of the resulting vulcanizates rather than the intrinsic wettability of the isolated BC. Direct contact angle measurements on biochar powders are difficult to interpret reliably because of the lack of a homogeneous and smooth surface and the possibility of partial penetration or absorption of the water droplet into the porous filler. The use of vulcanized samples provided a defined solid surface suitable for sessile-drop measurements and allowed the effect of BC modification to be evaluated under conditions relevant to the final rubber composites.

## 4. Conclusions

The obtained results demonstrate that walnut-shell biochar (BC) can be effectively used as a multifunctional filler for natural rubber (NR), although its performance strongly depends on the applied purification and surface-functionalization route. Chemical treatment with citric acid (CA) and NaOH substantially modified the mineral composition and surface chemistry of BC, as confirmed by elemental analysis and FTIR spectroscopy. These changes affected the filler–filler and filler–rubber interactions, which was reflected in the vulcanization behavior, crosslink density, mechanical properties and Payne effect. The incorporation of untreated BC increased the tensile strength from 14.1 ± 1.3 to 22.1 ± 1.2 MPa, while APTS functionalization resulted in the highest crosslink density, reaching 3.45 × 10^−5^ mol cm^−3^.

Particularly important was the effect of BC modification on thermo-oxidative aging resistance. Removal of mineral components, including Fe, Ca and Mg, was associated with changes in the stability of the NR network during aging. The functionalization route further influenced this behavior, with the highest aging coefficient (K = 0.89) obtained for the CA-treated and CAl-functionalized system. NaOH treatment was particularly effective in reducing filler–filler interactions, as indicated by the decrease in the Payne effect from 75.5% for untreated BC to 40.1%.

Overall, the results indicate that the final performance of BC-filled NR is determined by the combined effects of filler composition, surface chemistry and filler–network structure. No single modification route provided the most favorable results for all investigated properties. Chemical purification followed by appropriate surface functionalization therefore provides an effective strategy for tailoring BC–NR interactions and improving the functional properties and long-term stability of natural-rubber composites.

## Figures and Tables

**Table 1:** Content of selected elements in walnut shells and untreated and chemically modified biochar samples.

Sample	Content [%]								
	Cu	Zn	Mn	Fe	Cr	Ni	Ca	Co	Mg
Walnut shells	0.007	0.009	0.000	0.153	0.002	0.000	1.227	0.000	0.276
BC	0.002	0.006	0.002	0.233	0.008	0.001	2.321	0.000	0.516
BC_CA	0.002	0.005	0.001	0.186	0.006	0.001	0.654	0.000	0.154
BC_NaOH	0.002	0.003	0.001	0.094	0.002	0.001	1.532	0.000	0.421

**Table 2:** Thermogravimetric characteristics of untreated and modified biochars.

Filler	T_10_ [°C]	Z_25-600_ [%]	R_900_ [%]
BC	407	13.9	31.6
BC_APTS	165	21.4	25.1
BC_CAl	179	28.0	29.1
BC_CA	363	13.7	22.3
BC_CA_APTS	355	15.6	22.3
BC_CA_CAl	189	26.7	22.1
BC_NaOH	267	15.2	15.9
BC_NaOH_APTS	266	17.1	16,0
BC_NaOH_CAl	187	26.7	16.4

**Table 3:** Rheometric characteristics of the natural rubber compounds.

Sample	M_min_ [dNm]	M_max_ [dNm]	*Δ*M [dNm]
NR0	0.31	3.19	2.88
NR_BC	0.33	4.72	4.39
NR_BC_APTS	0.36	4.47	4.11
NR_BC_CAl	0.23	3.97	3.74
NR_BC_CA	0.32	4.35	4.03
NR_BC_CA_APTS	0.34	4.38	4.04
NR_BC_CA_CAl	0.29	3.66	3.37
NR_BC_NaOH	0.34	4.89	4.55
NR_BC_NaOH_APTS	0.39	4.49	4.10
NR_BC_NaOH_CAl	0.28	4.42	4.14

**Table 4:** DSC-derived vulcanization temperature range, apparent heat effect (dCp) and glass transition temperature (Tg).

Sample	Vulcanization Range [°C]	dCp [J/g]	Tg [°C]
NR0	158–211	9.47	−63.65
NR_BC	157–189	11.83	−62.65
NR_BC_APTS	159–187	4.44	−63.21
NR_BC_CAl	157–184	4.18	−63.26
NR_BC_CA	155–218	13.92	−63.50
NR_BC_CA_APTS	121–209	23.69	−64.02
NR_BC_CA_CAl	137–195	5.77	−64.50
NR_BC_NaOH	142–214	6.00	−65.34
NR_BC_NaOH_APTS	159–201	7.05	−63.69
NR_BC_NaOH_CAl	158–210	7.85	−63.19

**Table 5:** Effective crosslink density of the investigated vulcanizates.

Sample	ν_e_·10^−5^[mol/cm^3^]
NR0	2.35 ± 0.23
NR_BC	2.84 ± 0.32
NR_BC_APTS	3.45 ± 0.26
NR_BC_CAl	2.83 ± 0.14
NR_BC_CA	2.53 ± 0.22
NR_BC_CA_APTS	3.04 ± 0.33
NR_BC_CA_CAl	2.11 ± 0.24
NR_BC_NaOH	3.13 ± 0.31
NR_BC_NaOH_APTS	3.23 ± 0.25
NR_BC_NaOH_CAl	3.00 ± 0.26

**Table 6:** Static mechanical properties of the vulcanizates.

Sample	SE_300_ [MPa]	TS [MPa]	EB [%]
NR0	1.79 ± 0.2	14.1 ± 1.3	708 ± 20
NR_BC	2.47 ± 0.2	22.1 ± 1.2	734 ± 9
NR_BC_APTS	3.44 ± 0.3	20.1 ± 0.3	600 ± 6
NR_BC_CAl	2.24 ± 0.2	21.6 ± 0.2	717 ± 4
NR_BC_CA	2.53 ± 0.2	16.3 ± 1.0	718 ± 8
NR_BC_CA_APTS	3.20 ± 0.2	17.7 ± 1.0	710 ± 12
NR_BC_CA_CAl	2.18 ± 0.1	17.1 ± 0.2	753 ± 7
NR_BC_NaOH	2.67 ± 0.2	20.1 ± 1.1	713 ± 18
NR_BC_NaOH_APTS	3.92 ± 0.3	20.0 ± 1.3	633 ± 2
NR_BC_NaOH_CAl	2.42 ± 0.2	18.7 ± 0.7	717 ± 18

**Table 7:** Storage modulus at low and high strain amplitudes (G′low and G′high), modulus decrease (ΔG′), and Payne effect of the NR/BC compounds.

Sample	G′Low [kPa]	G′High [kPa]	*Δ*G′ [kPa]	Payne Effect [%]
NR_BC	157.6 ± 0.2	38.6 ± 1.0	119.0	75.5
NR_BC_APTS	153.1 ± 0.6	44.5 ± 0.9	108.5	70.9
NR_BC_CAl	143.1 ± 0.4	50.3 ± 1.2	92.8	64.8
NR_BC_CA	128.6 ± 0.4	61.9 ± 0.8	66.7	51.8
NR_BC_CA_APTS	125.9 ± 0.1	64.3 ± 1.1	61.7	49.0
NR_BC_CA_CAl	157.2 ± 0.6	6.7 ± 0.4	150.5	95.8
NR_BC_NaOH	129.1 ± 0.3	77.3 ± 0.8	51.8	40.1
NR_BC_NaOH_APTS	176.7 ± 0.1	89.2 ± 0.5	87.5	49.5
NR_BC_NaOH_CAl	155.3 ± 0.3	46.2 ± 1.8	109.1	70.3

## Data Availability

The original contributions presented in this study are included in the article. Further inquiries can be directed to the corresponding author.
